# Categorized contrast enhanced mammography dataset for diagnostic and artificial intelligence research

**DOI:** 10.1038/s41597-022-01238-0

**Published:** 2022-03-30

**Authors:** Rana Khaled, Maha Helal, Omar Alfarghaly, Omnia Mokhtar, Abeer Elkorany, Hebatalla El Kassas, Aly Fahmy

**Affiliations:** 1 Cairo University, National Institute of Cancer, Radiology Department, Cairo, 11796 Egypt; 2 Cairo University, Computers and Artificial Intelligence, Computer Science Department, Cairo, 12613 Egypt

**Keywords:** Breast cancer, Cancer imaging, Computer science

## Abstract

Contrast-enhanced spectral mammography (CESM) is a relatively recent imaging modality with increased diagnostic accuracy compared to digital mammography (DM). New deep learning (DL) models were developed that have accuracies equal to that of an average radiologist. However, most studies trained the DL models on DM images as no datasets exist for CESM images. We aim to resolve this limitation by releasing a Categorized Digital Database for Low energy and Subtracted Contrast Enhanced Spectral Mammography images (CDD-CESM) to evaluate decision support systems. The dataset includes 2006 images, with an average resolution of 2355 × 1315, consisting of 310 mass images, 48 architectural distortion images, 222 asymmetry images, 238 calcifications images, 334 mass enhancement images, 184 non-mass enhancement images, 159 postoperative images, 8 post neoadjuvant chemotherapy images, and 751 normal images, with 248 images having more than one finding. This is the first dataset to incorporate data selection, segmentation annotation, medical reports, and pathological diagnosis for all cases. Moreover, we propose and evaluate a DL-based technique to automatically segment abnormal findings in images.

## Abstract (summary)

**Table:** 

Measurement(s)	Dual-Energy Contrast-Enhanced Digital Spectral Mammography
Technology Type(s)	digital curation
Sample Characteristic - Organism	Homo sapiens • Breast
Sample Characteristic - Location	Egypt

## Background & Summary

**Fig. 1:**
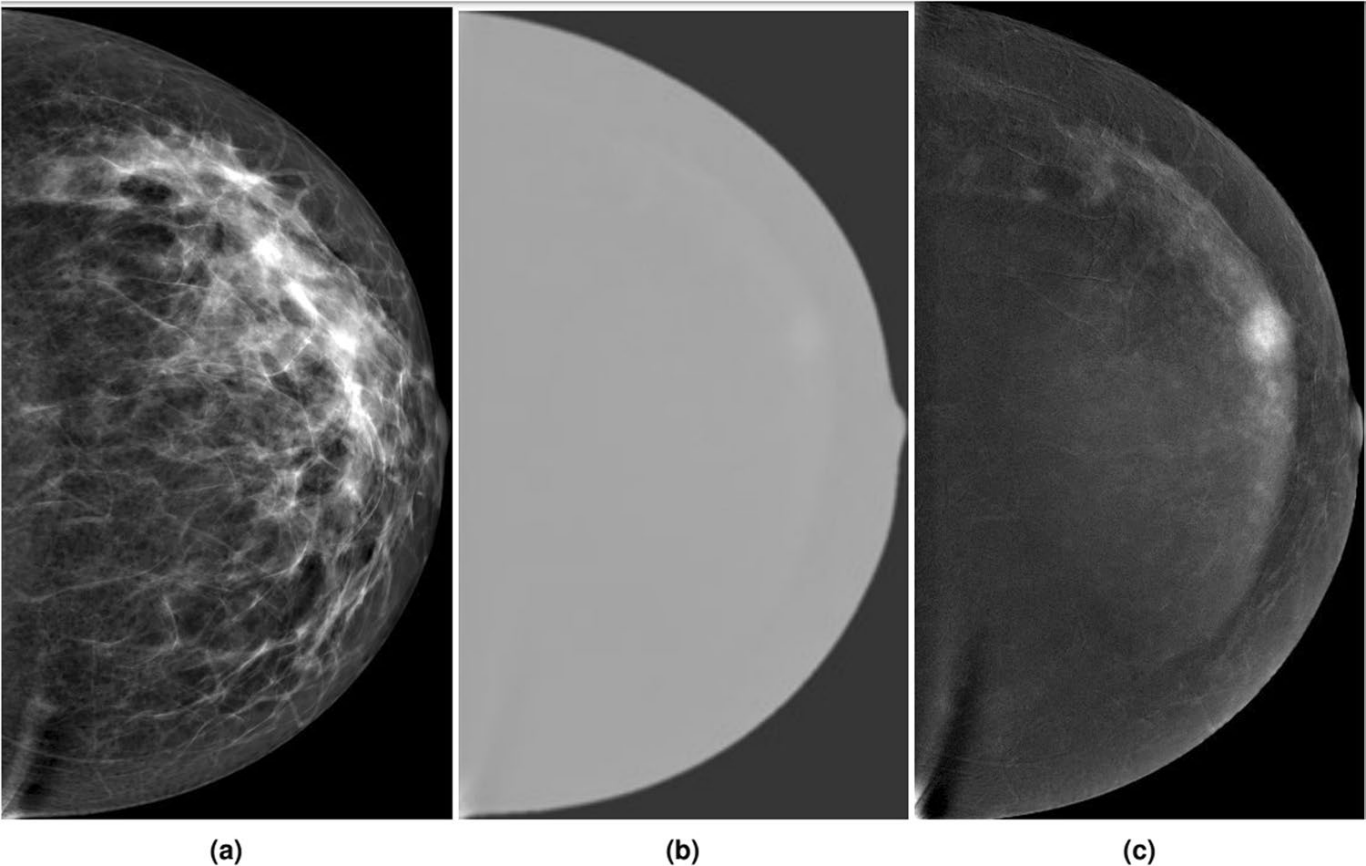
(**a**) Low-energy, (**b**) High-energy, and (**c**) Subtracted image.

Digital mammography (DM) is the gold standard imaging modality for early detection of breast cancer. However, limitations exist in patients with dense breasts as its overall sensitivity decreases^1^. Contrast-enhanced spectral mammography (CESM) is a contrast-based digital mammogram that has been approved by the Food and Drug Administration (FDA) in 2011 to be used as an adjunct to DM and ultrasound examinations for localization and characterization of occult or inconclusive lesions. Dual-energy image acquisition is performed where low and high-energy images are obtained. Several studies proved that low-energy images obtained appear like the standard DM images and are non-inferior to them^2^. High-energy images are non-interpretable; to overcome this, low and high-energy images are recombined and subtracted through appropriate image processing to suppress the background breast parenchyma after the acquisition. Figure 1 shows the resulting subtracted images obtained for interpretation, revealing contrast enhancement areas in a suppressed breast tissue background. Findings could be identified according to their density, morphologic, and enhancement characteristics^3^. However, estimating whether a lesion is benign or malignant without being seen by a radiologist is challenging due to the significant variation in the lesions’ visual characteristics^4^.

Computer-aided detection (CAD) systems were introduced in the early 2000’s to help radiologists interpret mammography images. However, this proved to be challenging in clinical practice due to the increased rate of false positives marked by the CAD systems, which can distract the radiologists^5^. Currently, the use of artificial intelligence (AI) in radiology is still in its early stages. Nonetheless, algorithms that analyze pixel data distinguish patterns from images that might not have been previously identified even by expert radiologists^6^. Deep learning (DL) has a promising potential in performing many tasks such as automatically detecting lesions and helping radiologists provide a more accurate diagnosis. Moreover, new multimodal DL models like the perceiver^7^ make it feasible to train on large datasets and extract good unsupervised image representations that can be used on a wide range of tasks. However, fully annotated and large-sized datasets are required and will be crucial for training new DL networks or fine-tuning existing pre-trained DL networks and evaluating them. This is why it is important for radiologists to understand the impact of these machine-learning (ML) based analytical tools and recognize how they might influence and change the radiological practice soon^8^.

In the past couple of years, a small number of public mammography datasets were released, including the Digital Database for Screening Mammography (DDSM)^9^, the Image Retrieval in Medical Applications (IRMA) project^10^, the Mammographic Imaging Analysis Society (MIAS) database^11^, and the Curated Breast Imaging Subset of DDSM (CBIS-DDSM)0^12^. These datasets contain DM images only, and none include CESM images.

In this paper, we present a CESM categorized dataset that provides easily-accessible low energy images with corresponding subtracted CESM images, abnormality segmentation annotation, verified medical reports, and pathological diagnosis for all cases. It will add to the ongoing advancements in future mammography DL-based systems. We also propose a new DL-based technique to automatically segment the abnormal findings in the images without intervention from radiologists, as segmentation annotation is a time-consuming task.

## Methods

**Fig. 2:**
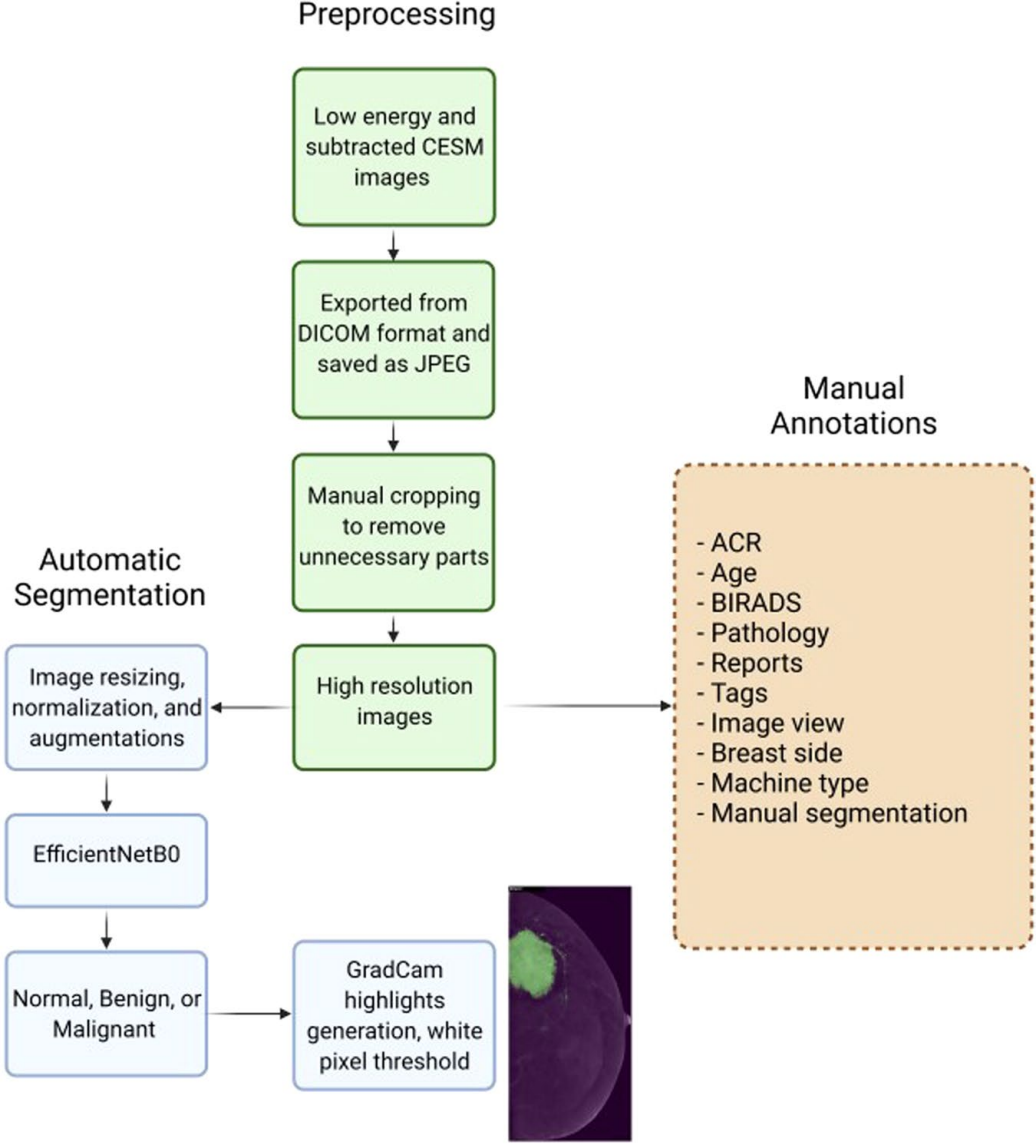
Flow diagram of the preparation of (CDD-CESM) and the deep learning method to automatically generate the segmentation annotation.

We collected and reformatted the data into an easily-accessible format. Figure 2 displays the flow diagram of the process to prepare our dataset: image preprocessing, manual annotations, and the automatic segmentation.

### Technique of contrast enhanced mammography examination

CESM is done using the standard DM equipment but with additional software that performs dual-energy image acquisition. Two minutes after intravenously injecting the patient with non-ionic low-osmolar iodinated contrast material (dose: 1.5 mL/kg), craniocaudal (CC) and mediolateral oblique (MLO) views are obtained. Each view comprises two exposures, one with low energy (peak kilo-voltage values ranging from 26 to 31kVp) and one with high energy (45 to 49 kVp). A complete examination is carried out in about 5–6 minutes.

### Description of dataset

**Fig. 3:**
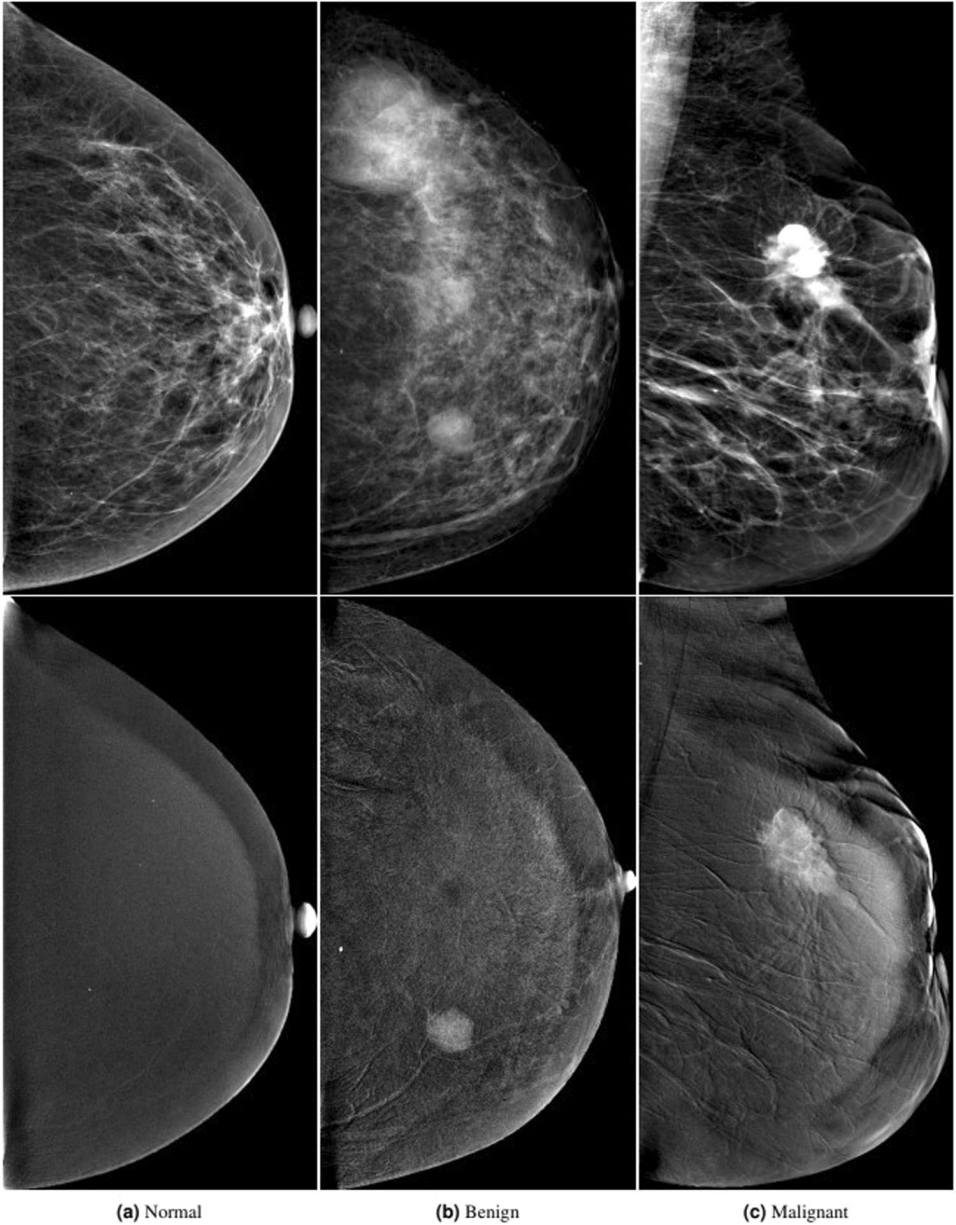
Samples of low energy and subtracted CESM images from the dataset.

The dataset is a collection of low-energy images with their corresponding subtracted CESM images gathered from the Radiology Department of the National Cancer Institute, Cairo University, Egypt over the period from January 2019 to February 2021. The images are all high resolution with an average of 2355 × 1315 pixels. Institutional review board approval and patient informed consent to carry out and publish data were obtained from 326 female patients aged from 18 to 90 years. The dataset contains 2006 images with CC and MLO views (1003 low energy images and 1003 subtracted CESM images), samples of low energy and subtracted CESM images are shown in Fig. 3. Usually, each patient has a total of 8 images, 4 images for each breast side consisting of low energy and subtracted CESM images for each CC and MLO view. However, there are 46 patients with only 4 images as they had mastectomy on a breast side, and 87 patients with missing images as some were not available or removed due to quality concerns. Two different machines were used for image acquisition; GE Healthcare Senographe DS and Hologic Selenia Dimensions Mammography Systems. The two machines provide similar quality, and all other steps in the data acquisition and post-processing phases were kept the same. The images are manually-annotated by expert radiologists according to the American College of Radiology Breast Imaging Reporting and Data System (ACR BIRADS) 2013 lexicon for standardized descriptors^13^. The annotations, shown in Table 1, include breast composition, mass shape, mass margin, mass density, architectural distortion, asymmetries, calcification type, calcification distribution, mass enhancement pattern, non-mass enhancement pattern, non-mass enhancement distribution, and overall BIRADS assessment (1 to 6). Both follow-up and pathological results are also included in the annotations, as pathological results are the gold-standard reference for radiologically-suspicious or malignant-looking lesions, and follow-up is the gold standard for benign-looking lesions. Moreover, full medical reports, written by an ensemble of radiologists, are provided for each case along with manual segmentation annotation for the abnormal findings in each image.

**Table 1:** Descriptions of the annotations available for the dataset.

Annotation	Description	Method	Format
Patient’s age	Age of the patient at time of examination.	Calculated from the date of birth.	Numbers
Side of breast	Right or left breast	Manually annotated.	Categorical
Breast Composition ACR category	Breast density describes the amount of fibroglandular tissue present in a breast relative to fat.	Blinded evaluation by two radiologists.	Categorical:
			**a**: Almost entirely fatty breasts
			**b**: Scattered fibroglandular tissue
			**c**: Heterogeneously dense breasts
			**d**: Extremely dense breasts
ACR BIRADS lexicon for standardized descriptors	Radiological lexicon providing the standard descriptors for evaluation of breast findings.	Blinded evaluation by two radiologists.	Mass shape, margin, and density.
			Architectural distortion.
			Asymmetries.
			Calcification type, and distribution.
			Mass enhancement pattern.
			Non-mass enhancement pattern, and distribution
Overall BIRADS	Radiological lexicon providing the final assessment categories for evaluation of breast findings.	Blinded evaluation by two radiologists.	BIRADS 1: Normal examination
			BIRADS 2: Benign findings
			BIRADS 3: Probably benign findings <2% malignancy
			BIRADS 4: Suspicious >2 but <95% malignancy
			BIRADS 5: Highly suspicious of malignancy >95%
			BIRADS 6: Known biopsy-proven malignancy
Type of image view	Usually two standard views are acquired for each breast:	Manually annotated.	Categorical:
	• MLO: most important because it allows depiction of most of the breast’s tissues		• MLO
	• CC: reveals medial part and external lateral portion of the breast		• CC
Tags	Labels assigned as follows:	Manually assigned and annotated by radiologist.	Categorical set of 140 unique tags.
	• Standardized descriptors of ACR BIRADS 2013 lexicon		
	• Probable diagnosis		
	• Classification		
Machine label	Two different mammography machines were used.	Manually annotated.	Machine number 1 or 2.
Pathology results / follow-up	Three classes: normal, benign, and malignant.	Manually annotated.	Categorical:
			• Normal
			• Benign
			• Malignant

### Annotations

Data are gathered and stored in a DICOM format. Some irrelevant annotations that are not used for lesion identification and classification were removed, including the patient’s name, ID, date of the study, and the image series. Each image with its corresponding annotation was compiled into one comma-separated-value (CSV) file.

### Medical reports

Separate corresponding reports for the CESM images and the DM images are also included in the dataset. Each report consists of the findings, depicted for each breast side separately, written following the ACR BIRADS 2013 lexicon for standardized descriptors and reporting associated with the BIRADS category annotated for the case. All patients’ identification data were removed. We believe that releasing the full-text medical reports is important, as research studies concerned with radiology report-writing often struggle with the lack of full reports not being present in large datasets^14^.

### Image processing

DICOM images were exported losslessly to a joint photographic experts group (JPEG) format using RadiAnt DICOM viewer application(https://www.radiantviewer.com/). After automatically removing all irrelevant data from each image, around 30% of the images were manually cropped to eliminate all unused and irrelevant boundaries. Furthermore, the images are named as follows {patient number}_{breast side}_{image type}_{image view}; example ‘P1_L_CM_MLO’.

### Segmentation visual model

In this section, we describe our method to automatically segment the abnormal parts of the images. A deep learning model, EfficientNetB0, was trained to predict the overall diagnosis (Normal, Benign, Malignant). GradCam^15^ was used to generate highlights for the parts of the image that contributed to the model’s prediction. A threshold of the top 25% GradCam intensities is then used on the highlights to generate the segments. Furthermore, a threshold of the top 15% white pixels is used to further finetune the segmentations.

#### Preprocessing

The images were first resized to be 224 × 224 using interpolation and anti-aliasing. Then the images were normalized by subtracting from the mean and dividing by the standard deviation. Random image augmentations were also used like cropping, zooming, and horizontal flipping. Furthermore, we experimented with non-traditional data augmentation methods^16^ which uses generative adversarial networks (GANs) to generate new images. However, the generated images did not satisfy the experts, so only traditional data augmentations were used.

#### Model & training

An EfficientNetB0^17^, pre-trained on ImageNet^18^, was used as the starting model in our experiments. We finetuned the model by removing the final layer and adding a layer with three output classes (Normal, Benign, Malignant). All the weights are left to be fine-tuned during the training. Categorical cross-entropy was used as the loss function with Adam optimizer^19^ as shown in Eq. 1, where *CE*(*b*) is the cross entropy loss for batch *b*, *C* the number of classes, *N* the number of images in the batch, *y* is the ground-truth, and $\widehat{y}$ is the prediction. A batch size of 16 was used, a decaying learning rate of 1e-3, and a dropout layer^20^ with a drop probability of 0.8 on the final visual features was used before the classifier.

1 $$CE(b)=-\mathop{\sum }\limits_{c=1}^{C}\mathop{\sum }\limits_{i=1}^{N}{y}_{{i}_{c}}.log\;{\widehat{y}}_{{i}_{c}}$$

#### Highlights

After the model achieved a good accuracy on all the images, we used GradCam^15^ to get heatmaps representing the parts of the image that had the highest impact on the model’s decision. The heatmaps are traced back from the ground-truth class and not the predicted class. Moreover, we removed any highlights in the corners of the image as they are often present at the location of normal pectoral muscles.

#### Segmentation

**Fig. 4:**
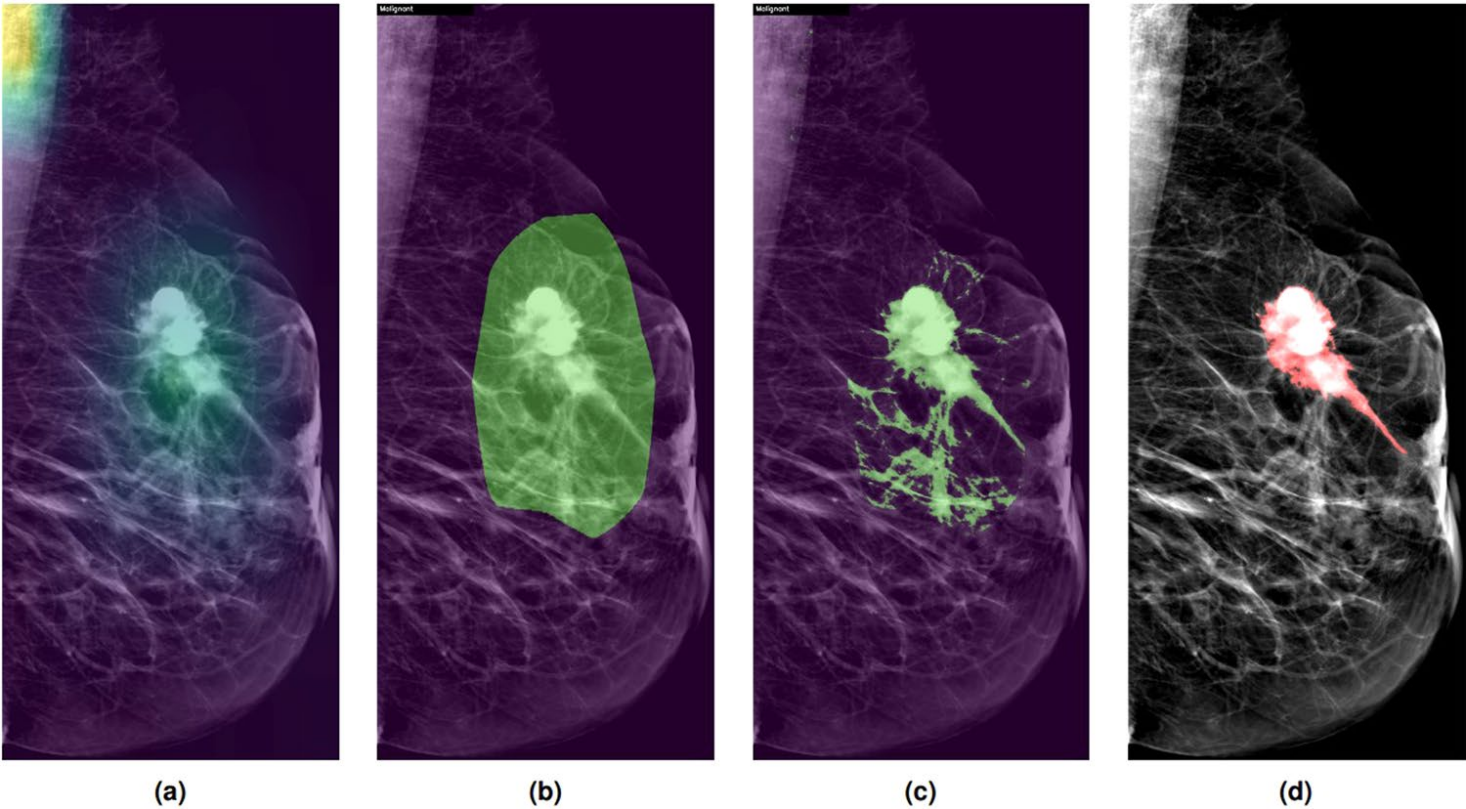
(**a**) Example of the DL Gradcam highlights, (**b**) Segmentation calculated after applying a threshold on the highlights, (**c**) Final output after applying the white pixel intensity threshold, and (**d**) Hand-drawn segmentation annotation.

To get the actual pixel segmentation, we used the top 25% of the heatmap’s intensities to serve as the abnormal segment. Moreover, to finetune the segments on the exact abnormality, we used the intersection of the segments and the top 15% white pixel intensities of the image as shown in Fig. 4.

## Data Records

The low energy and subtracted CESM images are distributed as JPEG files. They include both MLO and CC views of the mammograms.

Metadata for each image is incorporated as an associated CSV file consisting of:

- Path to image files
- Patient number
- Breast side: Left or Right
- Type of Examination: DM (low energy image) or CESM (subtracted image)
- View: CC or MLO
- Density category (if low energy image)
- Number of findings (if multiple)
- Mass shape, density, and margin (if present)
- Mass enhancement pattern (if present)
- Architectural distortion (if present)
- Asymmetry (if present)
- Calcification type and distribution (if present)
- Non-mass enhancement pattern and distribution (if present)
- BI-RADS assessment
- Pathology: Benign or Malignant

**Fig. 5:**
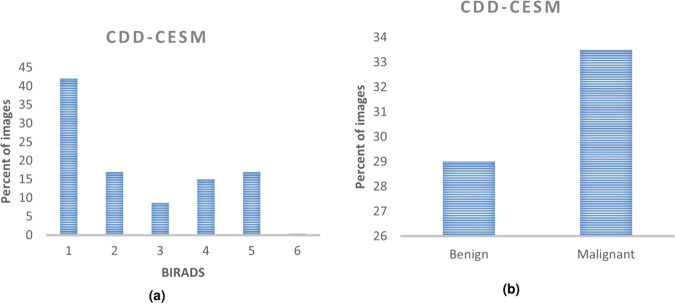
Histograms for the CDD-CESM dataset showing distribution of (**a**) BIRADS category for each abnormality, (**b**) Benign and malignant lesions.

Figure 5 shows histograms of BIRADS category and the corresponding final pathology/follow up result. Table 2 displays the characteristics of the CDD-CESM dataset.

**Table 2:** Characteristics of the CDD-CESM dataset.

		CDD-CESM
Stats	Years	2019–2021
	Sources	NCI, Cairo University
	No. females	326
	No. total images	2006
	No. normal images	757 (37.4%)
	No. benign images	587 (29.3%)
	No. malignant images	662 (33.3%)
Age (counted per patient)	<40	58 (17.8%)
	40–49	100 (30.7%)
	50–59	95 (29.1%)
	60–69	59 (18.1%)
	≥70	14 (4.3%)
Cancer Type	Invasive ductal carcinoma	445 (67.5%)
	Invasive lobular carcinoma	42 (6.3%)
	Mixed invasive ductal carcinoma and invasive lobular carcinoma	28 (4.2%)
	Ductal carcinoma insitu purely	17 (2.5%)
	Inflammatory breast cancer	40 (6%)
	Other	90 (13.5%)

The 757 normal images consist of 751 normal images and 6 post-neoadjuvant images considered normal (no residual disease proved by postoperative pathology). The age statistics are provided per number of patients.

The CDD-CESM dataset is available^21^ on The Cancer Imaging Archive repository^22^. The dataset includes all images, annotations, and full medical reports.

## Technical Validation

For the segmentation evaluation of our DL model, experienced radiologist provided hand-drawn segmentations for each abnormal finding in the CDD-CESM dataset. We calculated the intersection over union (IOU) and the dice coefficients (F1) between the computed and hand-drawn segmentations, after applying the same white-intensity threshold on the hand-drawn segmentations. Furthermore, we added another metric which we called overlap50, which is the percentage of images where the automatic segmentation overlaps with at least 50% of hand-drawn segmentation. The average IOU was 64.2% overall, overlap50 was 83.3%, and the average F1 was 71% overall. We also calculated these metrics separately for different groups of images according to the following criteria:

### Different findings represented in the dataset

Mass enhancement had the highest overlap50 = 91%. Furthermore, postoperative cases had the lowest overlap50 = 77%. This might be attributed to post operative edematous changes and skin thickening that are not accurately or completely observed by our DL model.

### Age of patient

Patients aged seventy years and higher had the highest overlap50 = 94%. Forty years and lower had the lowest overlap50 = 78%. As expected, the accuracy of visualization decreases as the breast density increases.

### Low energy or subtracted image

Low energy image overlap50 = 81%, compared to 86% in subtracted images. This might be due to the dense adenotic tissue in low-energy images obscuring abnormalities found behind it, which are suppressed in subtracted images. Thus, we recommend that radiologists use both low energy and subtracted images for each patient in each view, to increase reliability of using our DL technique in drawing their final conclusions.

### Mediolateral or Craniocaudal view

We found the results to be comparable without much difference in terms of automatic segmentation output.

### Benign or malignant finding

Benign findings had the lower overlap50 = 75% compared to 90% for malignant findings. Most of the benign lesions were non-enhancing in subtracted images. Furthermore, in low-energy images, benign lesions were either hidden behind the dense breast tissues, had equal density or parallel orientation to the surrounding breast parenchyma. However, highly cellular benign findings were accurately depicted by our DL model. Decreased accuracy was found with multiplicity and retroareolar locations.

**Fig. 6:**
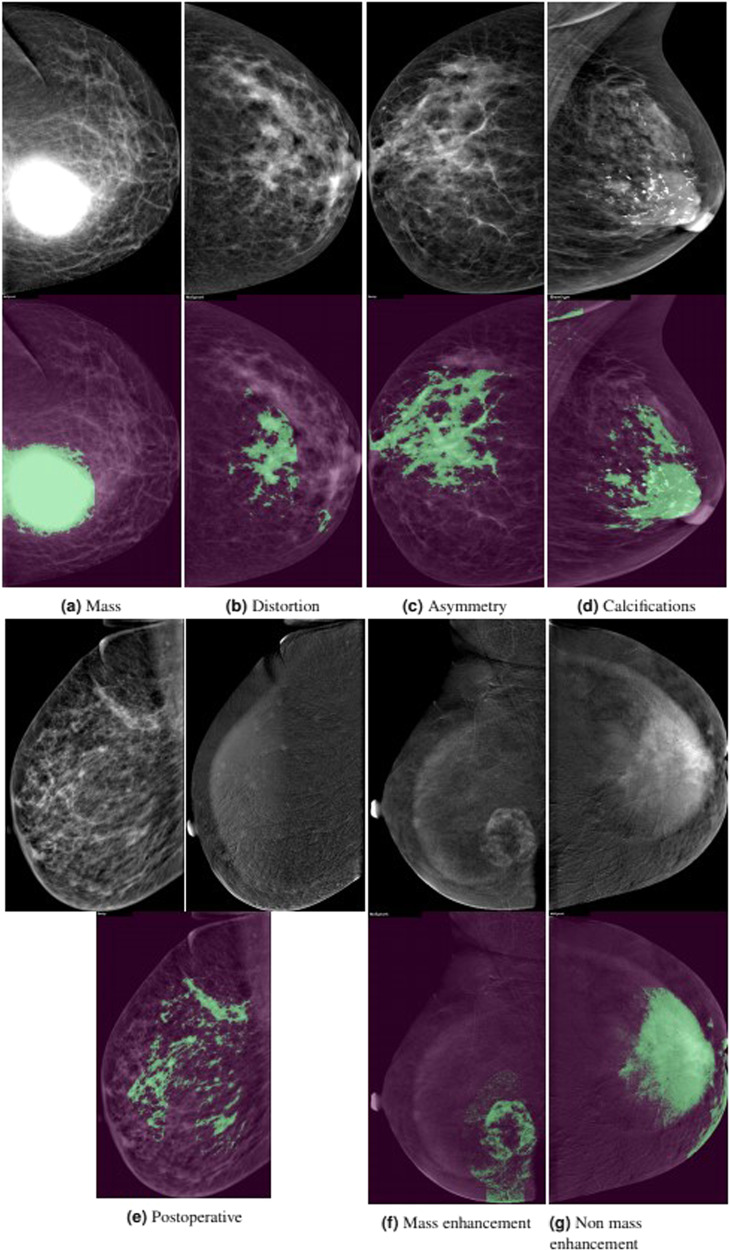
Examples of different cases and their corresponding automatic segmentations.

Generally, decreased accuracy of detection by our DL model was also present in some subtracted images with halo (breast-within-breast) or ripple artifacts. These calculations are shown in Table 3, and example outputs from our DL model are showed in Fig. 6.

**Table 3:** Detailed results of our DL segmentaion model.

		Images	Overlap50	IOU	F1
Findings	Mass	310	0.85	0.65	0.72
	Distortion	48	0.87	0.70	0.79
	Asymmetry	222	0.87	0.70	0.78
	Calcifications	238	0.81	0.62	0.70
	Postoperative	159	0.77	0.61	0.68
	Mass enhancement	334	0.91	0.66	0.73
	Non mass enhancement	184	0.89	0.72	0.79
Image Type	DM	665	0.81	0.64	0.71
	CM	590	0.86	0.65	0.71
Pathology	Benign	587	0.75	0.59	0.64
	Malignant	662	0.90	0.69	0.77
Image View	MLO	634	0.83	0.64	0.71
	CC	621	0.83	0.64	0.71
Machine	GE	1175	0.84	0.64	0.71
	Hologic	80	0.70	0.60	0.67
Age	<40	240	0.78	0.65	0.72
	40–69	958	0.83	0.64	0.70
	≥70	57	0.94	0.71	0.78

## Usage Notes

The dataset can be used to train machine learning models to classify mammogram images into normal, benign, and malignant, or classify the tags associated with each image. Moreover, it can be used to train segmentation models to segment the lesions. Furthermore, the full-text medical reports can be used to train report generation models.

## Data Availability

A Github repository is publicly available (https://github.com/omar-mohamed/CDD-CESM-Dataset) which contains helper scripts to make training a DL model on the dataset easier like reading the annotations, pre-processing the images by resizing and normalizing, training different existing models, augmenting the images while training, and evaluating the different models and plotting the segmentation results. The scripts were written using Python 3.6 with Tensorflow 2.3 for the training process, and OpenCV 4.1 and Pillow 6.1 for the image processing.
